# Clinical, Serological, and Molecular Profile of Dengue Patients With Warning Signs During the 2024 Outbreak in Belo Horizonte, Brazil

**DOI:** 10.1002/jmv.70805

**Published:** 2026-01-15

**Authors:** Samille Henriques Pereira, Ana Paula Moreira Franco‐Luiz, Camila Tita Nogueira, Guilherme Otávio Varino Cornélio, Adelina Machado de Carvalho Nogueira, Vírginia Antunes de Andrade, Silvia Hees de Carvalho, Karen Cecília de Lima Torres, Vanessa Peruhype‐Magalhães, Olindo Assis Martins‐Filho, Jordana Grazziela Alves Coelho‐dos‐Reis, Andréa Teixeira‐Carvalho, Flávio Guimarães da Fonseca, Pedro Augusto Alves

**Affiliations:** ^1^ Laboratório de Virologia Básica e Aplicada, Departamento de Microbiologia Universidade Federal de Minas Gerais Belo Horizonte Minas Gerais Brazil; ^2^ Laboratório de Imunologia de Doenças Virais Instituto René Rachou, Fiocruz Minas Belo Horizonte Minas Gerais Brazil; ^3^ Laboratório de Diagnóstico e Vigilância de Vírus Emergentes e Outros Patógenos Fiocruz Minas Belo Horizonte Minas Gerais Brazil; ^4^ Serviço de Doenças Infecciosas e Parasitárias Hospital Eduardo de Menezes, Fundação Hospitalar do Estado de Minas Gerais Belo Horizonte Minas Gerais Brazil; ^5^ Grupo Integrado de Pesquisas em Biomarcadores Instituto René Rachou, Fiocruz Minas Belo Horizonte Minas Gerais Brazil

**Keywords:** clinical progression, dengue virus, epidemiology, molecular diagnosis, platelet count, serological diagnosis

## Abstract

Dengue poses a significant arboviral threat in Brazil, with 2024 recording the largest outbreak to date. This prospective observational study was conducted during the 2024 outbreak with unvaccinated patients at Eduardo de Menezes Hospital, Belo Horizonte. A total of 556 patients were included, of whom 169 had complete clinical and laboratory data. Patients with suspected dengue underwent clinical and hematological evaluations, as well as diagnosis by RT‐qPCR, and ELISA. These parameters were employed to assess the relationship between diagnostic methods, hematological changes, and disease severity. RT‐qPCR confirmed dengue infection in 60% of with clinical symptoms, with partial overlap between PCR positivity and IgM detection, reflecting time‐dependent diagnostic windows. High IgG seropositivity indicated widespread prior exposure in the population. Thrombocytopenia was the most consistent hematological finding, with platelet counts declining until day 8 of symptoms and recovering around day 10; a secondary decline was observed in some patients with prolonged hospitalization. No significant differences in clinical severity were observed across serotypes, although DENV‐2 showed a trend toward lower platelet counts. These findings highlight the importance of integrating molecular and serological diagnostics during outbreaks and reinforce platelet monitoring as a key parameter for identifying patients at risk of severe dengue.

## 1 Introduction

Dengue is an arboviral disease of significant public health importance, particularly in tropical and subtropical regions. Its etiological agent is the Dengue virus (DENV ‐ *Orthoflavivirus denguei*), a virus transmitted by mosquitoes of the *Aedes* genus, which belong to the *Flaviviridae* family. This family also includes other medically significant viruses, such as yellow fever virus (YFV ‐ *Orthoflavivirus flavi*), Zika virus (ZIKV ‐ *Orthoflavivirus zikaense*), and West Nile virus (WNV ‐ *Orthoflavivirus nilense*) [1].

In 2024, Brazil experienced a new dengue outbreak, with 6,601,253 probable cases and a predominance of DENV‐1 and DENV‐2 serotypes [2]. However, a significant increase in cases caused by DENV‐3 was observed. The last epidemic associated with DENV‐3 occurred in 2008; sustained circulation has not been reported since then. Between 2010 and 2020, approximately 100 DENV‐3 cases were documented across the country, whereas over 1000 cases were confirmed solely in 2024, highlighting its reemergence in the national epidemiological context [3, 4, 5]. Minas Gerais was the Brazilian state that reported the highest number of dengue cases in 2024, with more than 1,695,098 probable cases and 1,374,633 confirmed cases [6]. Belo Horizonte, the state capital, reported 208,797 confirmed cases, corresponding to approximately 10% of its total population [7, 8].

Patients showing warning signs for dengue are particularly of concern, as they can progress to severe forms of the disease, such as dengue hemorrhagic fever and dengue shock syndrome. The warning signs include intense and persistent abdominal pain, bleeding, respiratory distress, hypotension, increased hematocrit, and a decrease in platelet count [9, 10, 11]. Active surveillance and continuous monitoring of these patients are essential to reduce mortality and morbidity associated with dengue [11]. Early detection of these signs, coupled with laboratory confirmation by RT‐PCR and ELISA, is critical for proper clinical management [12]. This study describes a prospective observational analysis of clinical and laboratorial findings of the cases of dengue with warning signs admitted to the Eduardo de Menezes Hospital (Belo Horizonte, Minas Gerais, Brazil) during the 2024 outbreak with unvaccinated patients, highlighting the supportive diagnosis and the correlation between clinical data and laboratory results to understand the clinical‐laboratory profile of the affected patients.

## 2 Methods

### 2.1 Study Design

The study investigated hospitalized patients with warning signs admitted to Eduardo de Menezes Hospital between March and May 2024, which corresponds to the period with the highest incidence of cases in Brazil. Blood samples were collected on consecutive days of hospitalization, processed for serum separation, and forwarded to the Laboratório de Diagnóstico e Vigilância de Vírus Emergentes e Outros Patógenos ‐ VigiLab (Fiocruz Minas) for differential molecular diagnosis of DENV (1–4) and other arboviruses, and to the Laboratório de Virologia Básica e Aplicada (Universidade Federal de Minas Gerais) for ELISA testing. Clinical severity was classified at admission according to the Brazilian Ministry of Health dengue risk classification, which is aligned with the World Health Organization (WHO) criteria. Patients were categorized as: A ‐ No spontaneous or induced bleeding (negative lasso test), no warning signs, no special condition, no social risk, and no comorbidities (WHO classification – dengue without warning signals). B ‐ With spontaneous or induced skin bleeding (lasso test +), or special clinical condition, social risk, or comorbidities, and no warning signs (WHO classification – dengue without warning signals with risk factors). C ‐ Presence of some warning sign. Hemorrhagic manifestation present or absent (WHO classification – dengue with warning signals). D ‐ With signs of shock. Respiratory distress; severe hemorrhage; severe organ dysfunction. Hemorrhagic manifestation present or absent (WHO classification – several dengue) [13, 14]. Clinical and demographic data, including hematological parameters, admission classification categories, age, sex, and geographic location, among others, were obtained from the hospital records in compliance with the Brazilian General Data Protection Law. The investigation was approved by the Ethics and Research Committee of René Rachou Research Center (Fiocruz Minas) (CAAE: 79060024.8.0000.5091).

### 2.2 Molecular Assay (RT‐qPCR)

RNA was extracted from serum samples using the automatic extractor Extracta 96 (Loccus, Brazil). Subsequently, the Trioplex real‐time RT‐PCR assay [15] was as used for amplification and detection of RNA from ZIKV, DENV serotypes, and the Chikungunya virus (CHIKV ‐ *Alphavirus chikungunya*). A homemade methodology was used to detect RNA from Oropouche virus (OROV ‐ *Orthobunyavirus oropoucheense*) and Mayaro virus (MAYV ‐ *Alphavirus mayaro*), employing primers described by Naveca et al., 2017 [16].

### 2.3 Serological Assay

For serological evaluation, ELISA was performed on patient sera using the Panbio™ Dengue IgM Capture ELISA kit (Abbott, EUA) for IgM detection and the Euroimmun Dengue Virus IgG ELISA kit (Euroimmun, Germany) for IgG detection. All assays were conducted according to the manufacturer's instructions. Positive and negative controls, as well as calibrators, were included on each plate. The capture format was used for IgM detection to reduce interference from pre‐existing IgG antibodies, while the indirect format was employed for IgG detection, as commonly applied for assessing prior exposure and secondary immune responses in dengue‐endemic settings [17]. The optical density (OD) ratio of each control and sample relative to the calibrator was calculated. Results were interpreted as negative (OD ratio < 0.8), borderline (0.8 ≤ OD ratio < 1.1), or positive (OD ratio ≥ 1.1).

### 2.4 Statistical Analyses

All statistical analyses were performed using GraphPad Prism version 8.0.2. Categorical variables were compared between dengue serotypes and clinical groups using the Chi‐square test or Fisher's exact test, as appropriate. Continuous variables were assessed for normality using the Shapiro‐Wilk test. Normally distributed data were compared using Student's *t*‐test or one‐way ANOVA, while non‐normally distributed data were analyzed using the Mann–Whitney or Kruskal–Wallis tests. A *p*‐value of < 0.05 was considered statistically significant. Results are presented as mean ± standard deviation or median (interquartile range), depending on data distribution.

## 3 Results

### 3.1 Clinical, Serological, and Molecular Findings in the Study Cohort

A total of 556 patients with warning signs of dengue were hospitalized at Eduardo de Menezes Hospital (Belo Horizonte, Minas Gerais, Brazil) between March and May 2024. Due to the overload of the healthcare system during the outbreak, hospitalization was based on clinical symptoms, without prior laboratory confirmation.

**Figure 1:**
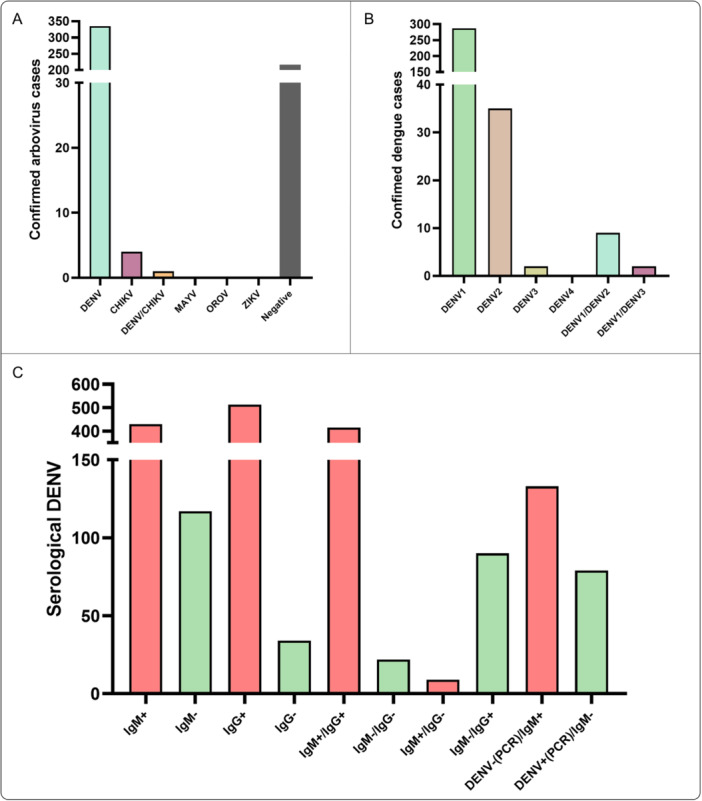
Molecular and serological characteristics of hospitalized dengue patients. Serum samples collected at hospital admission were tested by RT‐qPCR for DENV (serotypes 1–4), CHIKV, ZIKV, MAYV, and OROV, and by ELISA for DENV‐specific IgM and IgG. (A) Proportion of patients testing positive for DENV, CHIKV, and DENV/CHIKV coinfection, as well as RT‐qPCR–negative cases. (B) Distribution of DENV serotypes among RT‐qPCR–positive patients, highlighting the predominance of DENV‐1 and the detection of DENV‐2, DENV‐3, and mixed infections (DENV‐1/DENV‐2 and DENV‐1/DENV‐3). No DENV‐4 cases were identified. (C) Serological profiles based on IgM and IgG ELISA results, including single‐ and dual‐antibody positivity and their relationship with RT‐qPCR outcomes, illustrating partial overlap between molecular and serological diagnostic windows.

RT‐qPCR assays were performed for DENV (serotypes 1–4), ZIKV, CHIKV, MAYV, and OROV. DENV was detected in 60.2% (*n* = 335) of patients, CHIKV in 0.7% (*n* = 4), and DENV/CHIKV coinfection in 0.1% (*n* = 1), while 38.8% (*n* = 216) tested negative. No cases of ZIKV, MAYV, or OROV were identified (Figure 1A). Among DENV‐positive cases, serotyping revealed a predominance of DENV‐1 (85.7%, *n* = 287), followed by DENV‐2 (10.5%, *n* = 35) and DENV‐3 (0.6%, *n* = 2). Coinfections of DENV‐1/DENV‐2 (2.6%, *n* = 9) and DENV‐1/DENV‐3 (0.6%, *n* = 2) were also detected, whereas no cases of DENV‐4 were detected (Figure 1B).

Serological testing showed that 77.5% (*n* = 429) of patients were positive for DENV‐specific IgM and 92% (*n* = 513) were positive for IgG. Concomitant IgM and IgG positivity was observed in 75% (*n* = 415), while 4% (*n* = 22) were negative for both antibodies. IgM positivity with IgG negativity was detected in 1.5% (*n* = 9), whereas IgG positivity with IgM negativity occurred in 16% (*n* = 90). When comparing serology with molecular testing, 24% (*n* = 133) of patients with negative DENV RT‐qPCR results were IgM positive, while 14% (*n* = 79) of PCR‐positive patients were IgM negative (Figure 1C). Patients negative for IgM and PCR represent 2% (*n* = 13). A significant association was observed between DENV RT‐qPCR results and IgM seropositivity (χ² = 13.94; df = 1; *p* = 0.00019). Among PCR‐positive patients, 76.4% were IgM positive versus 61.6% among PCR‐negative patients, suggesting higher IgM detection coinciding with molecular positivity (reflecting overlapping diagnostic windows).

**Figure 2:**
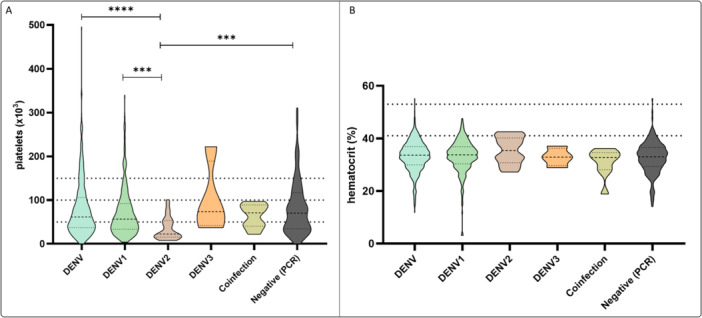
Hematological parameters of hospitalized patients with suspected dengue, stratified by DENV serotype and RT‐qPCR status. Platelet counts and hematocrit levels measured at hospital admission were compared across patients with different DENV serotypes and those who tested negative by RT‐qPCR. Thrombocytopenia (< 150,000/µL) was highly prevalent, occurring in 89% of patients, while elevated hematocrit (> 43%) was observed in a small subset (2.3%). The figure displays the distribution of platelet counts and hematocrit values, highlighting significant differences in platelet medians between DENV‐2 cases and both DENV‐1 and PCR‐negative patients.

Clinical laboratory parameters were also evaluated. Platelet counts and hematocrit levels at admission were analyzed to assess hematological changes associated with dengue infection. Thrombocytopenia (platelets < 150,000/µL) was observed in 89% (*n* = 497) of patients, whereas elevated hematocrit levels (> 43%) occurred in 2.3% of the study population (*n* = 13). A statistically significant difference was observed in platelet median counts between DENV‐2 and both DENV‐1 and negative cases. Distributions of platelet counts and hematocrit values across different DENV serotypes and PCR status are presented in Figure 2.

### 3.2 Follow‐Up of Patients with Comprehensive Clinical Data

A subset of 169 patients from the overall cohort had complete clinical and demographic data available and were included in this detailed analysis. This subset contains information on age, sex, initial and final dengue classification, comorbidities, and hematological parameters at admission. The main symptoms reported were abdominal pain, nausea, vomiting, fever, postural hypotension, respiratory distress, and mucosal bleeding. Table 1 summarizes the baseline characteristics and hematological profiles of these patients.

**Table 1:** Distribution of Patients with Warning Signs for DENV by Demographic Characteristics, Comorbidities, and Hematological Parameters.

	DENV suspected *n *= 169	DENV1 *n* = 116 (68.7)	DENV2 *n* = 9 (5.4)	DENV3 *n* = 1 (0.6)	DENV1/DENV2 *n* = 7 (4.0)	NEG *n* = 36 (21.3)
Age						
0–17	7 (4.1)	6 (85.7)	—	—	—	1 (1.3)
18–64	114 (67.4)	76 (66.6)	7 (6.1)	1 (0.8)	7 (6.1)	23 (20.4)
≥ 65	48 (28.5)	34 (70.8)	2 (4.2)	—	—	12 (25.0)
Sex						
Female	90 (53.3)	62 (68.9)	4 (4.4)	1 (1.1)	5 (5.6)	18 (20.0)
Male	79 (46.7)	54 (68.3)	5 (6.3)	—	2 (2.5)	18 (22.7)
Dengue Initial Classification						
A*	1 (0.6)	—	—	—	1 (100.0)	—
B*	33 (19.5)	22 (66.6)	1 (3.3)	—	3 (9.0)	7 (21.1)
C*	123 (72.7)	85 (69.1)	8 (6.5)	1 (0.8)	3 (2.4)	26 (21.2)
D*	7 (4.2)	5 (71.4)	—	—	—	2 (28.6)
N/A	5 (3.0)	4 (80.0)	—	—	—	1 (0.2)
Dengue Final Classification						
A*	33 (19.5)	27 (81.8)	—	—	—	6 (18.2)
B*	90 (53.3)	69 (76.7)	1 (1.1)	1 (1.1)	3 (3.3)	16 (17.8)
C*	14 (8.2)	1 (7.1)	8 (57.2)	—	3 (21.4)	2 (14.3)
Death	2 (1.2)	1 (50.0)	—	—	—	1 (50.0)
Transfer	2 (1.2)	1 (50.0)	—	—	—	1 (50.0)
N/A	28 (16.6)	17 (60.7)	—	—	1 (3.5)	10 (35.8)
Comorbidities						
Systemic Arterial Hypertension	64 (37.8)	42 (65.7)	5 (7.8)	—	1 (1.5)	16 (25.0)
Diabetes	26 (15.4)	16 (61.5)	2 (7.7)	—	1 (3.8)	7 (27.0)
Dyslipidemia	23 (13.6)	14 (61.0)	1 (4.3)	—	1 (4.3)	7 (30.4)
Heart Failure	10 (6.0)	8 (80.0)	—	—	—	2 (20.0)
Hypothyroidism	19 (11.2)	14 (73.7)	1 (5.3)	—	1 (5.3)	3 (15.7)
Depression	13 (7.7)	9 (69.2)	2 (15.4)	1 (7.7)	—	1 (7.7)
Others	99 (58.6)	74 (74.7)	4 (4.1)	—	4 (4.1)	17 (17.1)
Hematological profile						
*Platelets (mm* ^*3*^ *)*						
< 50,000	74 (43.8)	51 (69.0)	7 (9.5)	1 (1.3)	5 (6.7)	10 (13.5)
50,000–100,000	50 (30.0)	36 (72.0)	2 (4.0)	—	1 (2.0)	11 (22.0)
100,000–150,000	27 (16.0)	15 (55.6)	—	—	1 (3.7)	11 (40.7)
> 150,000	18 (10.2)	14 (77.7)	—	—	—	4 (22.3)
*Hematocrit (%)*						
< 41	106 (62.7)	72 (68.0)	7 (6.5)	1 (0.9)	5 (4.6)	21 (20.0)
41–43	15 (8.8)	11 (73.4)	—	—	2 (13.3)	2 (13.3)
> 43	48 (28.5)	33 (68.8)	2 (4.2)	—	—	13 (27.0)

*Note:* Values are expressed as absolute numbers and percentages.

* Risk classification according to the Brazilian Ministry of Health, aligned with WHO dengue classification criteria (see Materials and Methods for details).

Among the patients included in the analysis, the median age was 53 years (IQR: 32–65 years), with a 95% confidence interval for the median of 50–55 years. Most patients were between 18 and 64 years old (67.4%), followed by those ≥ 65 years (28.5%) and children/adolescents aged 0–17 years (4.1%). The demographic profile of the cohort subset, with a predominance of females (53.3%) and individuals aged 18–64 years (67.4%), was consistent with national data from the Ministry of Health Arbovirus Monitoring Panel for 2024, which reported 55% females and 70.2% aged 20–69 years, suggesting that our sample reflects the broader epidemiological context of dengue in Brazil [2].

Regarding laboratory diagnosis, DENV‐1 was the most frequently identified serotype, followed by DENV‐2 and DENV‐3, while a smaller proportion of samples tested negative for dengue. The most prevalent comorbidities were systemic arterial hypertension (37.8%), diabetes mellitus (15.4%), and dyslipidemia (13.6%), with other conditions reported less frequently.

Regarding the initial clinical classification, most patients were classified as dengue with warning signs (class C, 72.7%), followed by dengue without warning signs but with risk factors (class B, 19.5%), severe dengue (class D, 4.2%), and dengue without warning signals (class A, 0.6%). In the final classification, the majority remained in class B (53.3%), followed by classes A (19.5%), C (8.2%), death (1.2%), and transfer (1.2%).

Concerning the hematological profile, the median platelet count was 59 × 10³/µL (IQR: 30.5–103 × 10³/µL), with a 95% confidence interval for the median of 49–70 × 10³/µL, and the median hematocrit was 33.3% (IQR: 29.7–36.95%), with a 95% confidence interval of 32.5–33.9%. Consistent with the findings observed in the overall cohort, patients infected with DENV‐2 in the subset with complete clinical data presented lower platelet counts compared to those with DENV‐1.

**Figure 3:**
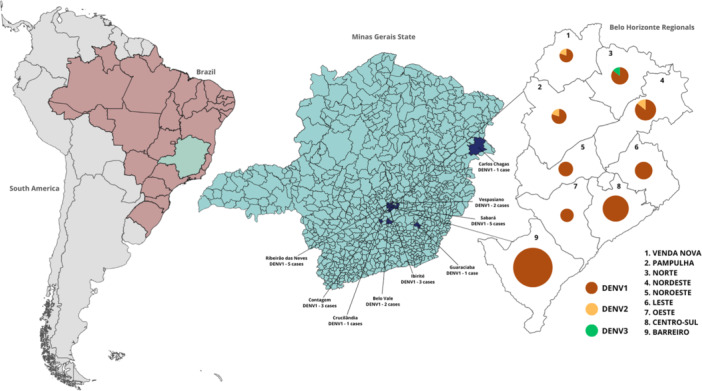
Geographical distribution of dengue patients presenting warning signs. In the city of Belo Horizonte, cases were classified according to administrative regions: Venda Nova (*n* = 5), Pampulha (*n* = 5), Norte (*n* = 7), Nordeste (*n* = 10), Noroeste (*n* = 6), Leste (*n* = 7), Oeste (*n* = 5), Centro‐Sul (*n* = 16), and Barreiro (*n* = 24). In the metropolitan area of Belo Horizonte (RMBH), cases were recorded in Vespasiano (*n* = 2), Sabará (*n* = 5), Ribeirão das Neves (*n* = 5), Contagem (*n* = 3), and Ibirité (*n* = 3). Cases from other municipalities in the state of Minas Gerais included Belo Vale (*n* = 2), Carlos Chagas (*n* = 1), Cruscilândia (*n* = 1), and Guaraciaba (*n* = 1). Geographical information was unavailable for the remaining 61 patients.

The Eduardo de Menezes Hospital is located in Belo Horizonte city, the capital of Minas Gerais state. As a reference center for infectious‐contagious diseases in the state of Minas Gerais, this hospital also receives cases from other municipalities in the metropolitan area and from some cities in the countryside. The spatial distribution of cases according to DENV serotype is shown in Figure 3. Most cases were concentrated in Belo Horizonte, with higher numbers in the Barreiro and Centro‐Sul regions, followed by the Nordeste and Leste regions (Figure 3). In the Belo Horizonte metropolitan area, cases were reported in municipalities such as Sabará, Ribeirão das Neves, and Contagem, while a smaller number of patients came from smaller countryside municipalities, including Carlos Chagas, Guaraciaba, and Belo Vale.

**Figure 4:**
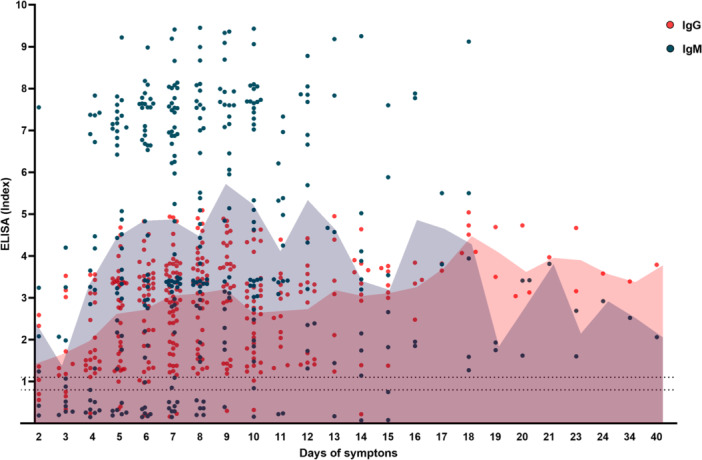
Temporal profile of IgM and IgG levels during hospitalization. Representation of the evolution of IgM (blue) and IgG (orange) antibody titers over the course of hospitalization. Symptom days are in accordance with patients’ reports. An early elevation of IgM was observed, with peak detection occurring near days 9–10 of illness, followed by a downward trend. IgG levels, however, continued to rise over time, a pattern compatible with secondary immune activation and suggestive of previous dengue infections among some individuals. These data reflect the overall serological response of the analyzed cohort, without distinction by diagnostic groups.

The serological analysis of patients revealed the temporal profile of antibodies against DENV during the first days of symptoms. IgM and IgG were evaluated in samples collected throughout the course of the disease. In the graph (Figure 4), each point represents an individual patient, while the filled lines indicate the daily mean of each marker. IgM rose early during the course of symptoms, peaking between days 9 and 10, followed by a gradual decline. IgG showed a progressive increase over time, reflecting a secondary immune response and suggesting previous exposure in some patients.

### 3.3 Clinical Profile: Disease Severity and Serotype

**Figure 5:**
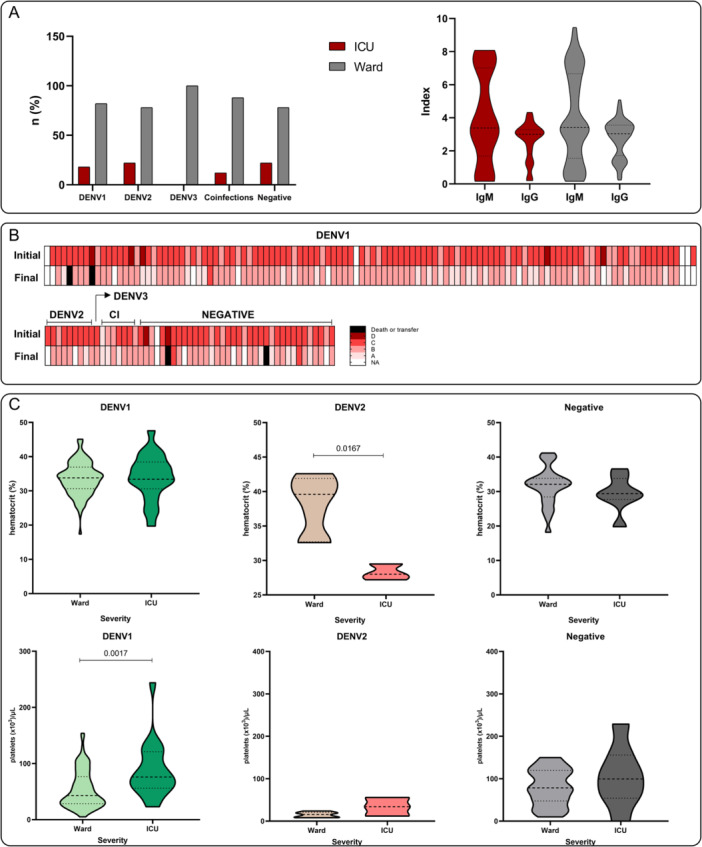
Clinical and laboratory characteristics of patients by dengue serotype. (A) Proportion of patients admitted to the ICU versus ward by dengue serotype and comparison of IgM and IgG indices between ICU and ward patients. (B) Evolution of clinical classification from initial to final assessment for each dengue serotype, showing overall improvement over time. (C) Laboratory parameters by dengue serotype and care setting.

At the time of hospital admission, a comparison of ICU (Intensive Care Unit) admission versus ward stays among patients by dengue serotype showed no statistically significant association (χ² = 4.44; df = 3; *p* = 0.217). The proportion of patients admitted to the ICU was similar across serotypes: 18% for DENV‐1, 22% for DENV‐2, 12% for coinfection, and 22% for PCR‐negative cases (Figure 5A). Similarly, no significant differences were observed in IgM and IgG indices between ICU and ward patients (Figure 5A). These findings suggest that neither the requirement for ICU care nor the humoral immune response, as measured by IgM and IgG indices, was significantly influenced by dengue serotype in this cohort.

The analysis of the evolution of clinical classification over time showed that most patients improved from their initial to final classification. When assessed separately by serotype, the same trend was observed. These results indicate that, although initial disease severity varied among patients, clinical progression did not differ significantly amongst serotypes (Figure 5B).

Analysis of laboratory parameters revealed specific differences amongst serotypes and locations of care. Platelet counts were significantly higher in ward patients with DENV‐1 compared to those in the ICU (Figure 5C). Hematocrit values were significantly different between ICU and ward patients with DENV‐2 (Figure 5C). No statistically significant differences in laboratory parameters were observed for the other serotypes between care settings.

**Figure 6:**
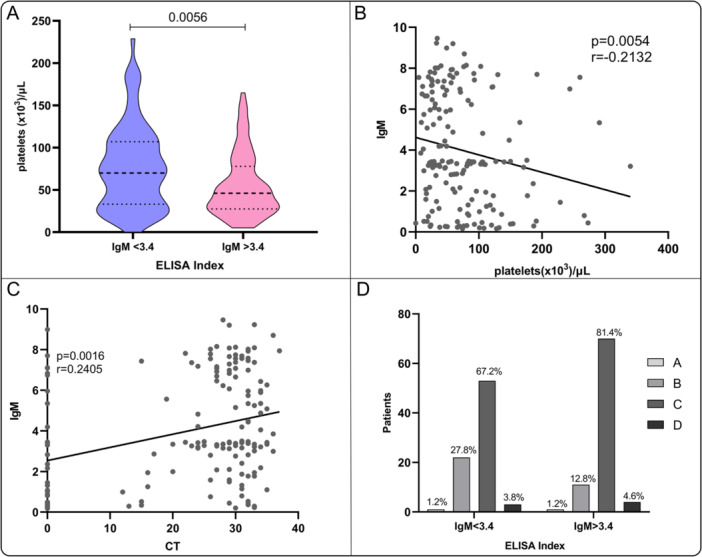
Correlation between anti‐DENV IgM levels, platelet counts, and viral load on the first day of hospitalization. (A) Comparison of patients with IgM indices above and below the median (3.4) in relation to platelet counts. Patients with higher IgM indices exhibited more pronounced thrombocytopenia compared to those with lower IgM levels. (B) Spearman's correlation analysis showing a significant inverse correlation between IgM levels and platelet counts (*r* = –0.2132, *p* = 0.0054). (C) Spearman's correlation analysis showing a significant positive correlation between IgM levels and CT values (*r* = 0.2405, *p* = 0.0016). Viral load was measured by RT‐qPCR and expressed as cycle threshold (CT) values, with lower CT values indicating higher viral loads. (D) Histogram showing the distribution of patients by initial dengue classification (A–D) stratified by IgM indices (< 3.4 vs > 3.4). Patients with higher IgM indices tended to present more frequently with severe dengue categories (C and D), whereas milder categories (A and B) were more common among patients with lower IgM indices. When grouped as mild (A + B) versus severe (C + D), this association was statistically significant (χ² = 5.7, *p* = 0.017).

An important observation was made regarding IgM indices, platelet counts, and viral load assessed on the first day of hospitalization. Patients with higher IgM indices exhibited lower platelet counts, indicating more pronounced thrombocytopenia, whereas those with lower IgM indices showed higher platelet counts (Figure 6A). Spearman's correlation analysis confirmed a significant inverse correlation between anti‐DENV IgM levels and platelet counts (*r* = –0.2132, *p* = 0.0054) (Figure 6B), and a significant positive correlation between IgM levels and cycle threshold (CT) values (*r* = 0.2405, *p* = 0.0016) (Figure 6C), consistent with an inverse association between IgM and viral load, given that CT is inversely related to viral load. Additionally, patients were stratified by initial dengue classification (A–D) according to IgM indices (< 3.4 vs > 3.4), showing a trend toward more severe categories among those with higher IgM. When patients were grouped according to disease severity and compared as mild (A + B) versus severe (C + D), this association reached statistical significance (χ² = 5.7, *p* = 0.017) (Figure 6D).

**Figure 7:**
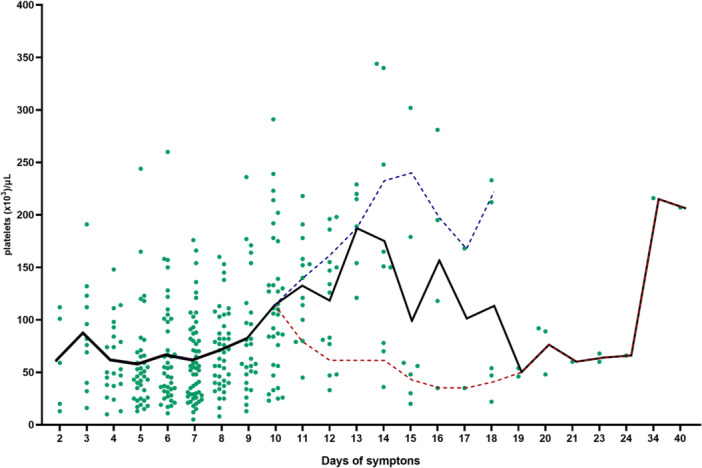
Longitudinal dynamics of platelets counts in hospitalized dengue patients. Each point represents an individual measurement per patient, while the line indicates the mean values by day of symptoms. A pronounced drop in platelets was observed until day 8, followed by recovery around day 10 (indicated by the dashed blue line). Subsequently, a secondary decline in platelet levels was observed in a subset of patients, particularly those with prolonged hospitalization and more severe disease (indicated by the dashed red line). Reference values for platelets: 150,000 a 400,000/mm^3^.

The longitudinal analysis of platelet counts revealed characteristic dynamics throughout the clinical course of hospitalized patients (Figure 7). A pronounced decline was observed until approximately the eighth day of symptoms, which coincides with the critical phase of dengue infection when warning signs and complications are more likely to appear. Around day ten, platelet counts showed a partial recovery, reflecting the beginning of the convalescent phase in many patients. Among patients infected with DENV‐2, platelet counts remained below 50,000/mm³ until approximately day 10 of symptom onset, followed by an increase to values around 100,000/mm³ between days 10 and 12, and recovery to levels above 150,000/mm³ after day 14. Patients with DENV‐2 and complete information on symptom duration could be followed for up to 15 days after symptom onset. These findings suggest a delayed platelet recovery in DENV‐2 infections, consistent with the more severe thrombocytopenia observed in cross‐sectional analysis. Nevertheless, in individuals with prolonged hospitalization and more severe clinical evolution, platelet values exhibited a second decline beyond this period, reinforcing their potential as an indicator of disease progression and severity.

## 4 Discussion

The analysis conducted in this study provides a unique snapshot of the clinical, laboratory, and epidemiological scenario of the 2024 dengue epidemic among patients with warning signs in Minas Gerais, Brazil. The outbreak was predominantly associated with DENV‐1, followed by a lower proportion of DENV‐2. Noteworthy was the reemergence of DENV‐3 after a prolonged period without significant circulation in the state. This finding is consistent with recent reports of DENV‐3 reintroduction in other regions of Brazil [4, 5, 6, 7, 8, 9, 10, 11, 12, 13, 14, 15, 16, 17, 18], highlighting the current epidemiological complexity driven by the co‐circulation of multiple serotypes [19]. Such viral diversity increases the risk of secondary infections and, consequently, the development of more severe disease, posing additional challenges to healthcare services and control strategies [20].

A study published by Rabelo et al. (2020), analyzing samples collected between 2009 and 2014, evaluated probable dengue cases using viral isolation and RT‐PCR. Among the confirmed cases, the authors reported the circulation of DENV‐1 (60%), DENV‐4 (22%), DENV‐2 (9.8%), and DENV‐3 (7.7%) [21]. This serotype distribution differs from the current scenario, as no DENV‐4 cases were detected in our study, consistent with findings reported in other Brazilian states in recent years. Published data from previous outbreaks in the same endemic region and more broadly in the state of Minas Gerais remain scarce, limiting direct temporal comparisons with earlier epidemics. Nevertheless, analyses of epidemiological reports issued by the Health Department Municipality of Belo Horizonte reveal a pattern that mirrors dengue dynamics observed in other regions of Brazil, characterized by cyclical epidemic peaks followed by periods of reduced transmission. Notably, large outbreaks occurred in 2016, 2019, and 2024, with the number of cases documented in 2024 exceeding all previously recorded years (Figure S1). When examining the serotypes detected in the endemic area over the past decade, DENV‐1 and DENV‐2 have been the most frequently identified (Figure S2), demonstrating their predominance in recent years, an epidemiological profile that is also reflected in the present study.

The comparison between molecular and serological assays demonstrated a significant association between RT‐qPCR positivity and IgM detection, reflecting partially overlapping diagnostic windows. This observation is particularly relevant in outbreak settings, where the choice of the most appropriate diagnostic test directly depends on the time of symptom onset [22]. Moreover, the high proportion of IgG‐positive patients suggests extensive prior circulation of the virus in the population, indicating that a subset of individuals could have mounted a secondary immune response [23]. Importantly, although dengue vaccination was introduced in Brazil in 2024, it was restricted to individuals aged 10–14 years, a group not included in this cohort. Therefore, IgG seropositivity in this study reflects prior natural infection rather than vaccine‐induced immunity.

Most patients in our cohort exhibited mild to moderate thrombocytopenia, reflecting the hematological impairment that is a hallmark of dengue virus infection [24, 25]. Although DENV‐1 was the predominant serotype detected, patients infected with DENV‐2 showed significantly lower platelet counts, supporting previous observations that this serotype is more frequently associated with marked thrombocytopenia and severe clinical outcomes [20, 26, 27]. Consistent findings were observed in the subset of patients with longitudinal follow‐up, in which platelet counts declined markedly until approximately the eighth day of symptoms, followed by a recovery around day ten. However, beyond this point, some patients experienced a subsequent decline in platelet levels, particularly those requiring prolonged hospitalization and presenting more chance of severe disease, a similar pattern when compared to other recent studies [28]. In contrast, hematocrit values remained relatively stable throughout the clinical course in most patients, with significant increases observed only in a minority of cases. While hematocrit is a well‐established marker of severity in dengue, particularly in the context of plasma leakage [29], its limited variation in our cohort may reflect early hospitalization and continuous intravenous fluid therapy.

Furthermore, while serotype coinfection was detected in a few cases, it occurred infrequently and did not appear to substantially alter the clinical or laboratory presentation, consistent with reports describing low prevalence and variable clinical significance of coinfections across regions [30].

No statistically significant differences were observed across serotypes regarding ICU admission or improvement in final clinical classification. Most patients showed clinical recovery, transitioning to less severe categories by the end of hospitalization, indicating that despite the serotype diversity and reemergence of DENV‐3, clinical outcomes remained favorable in this cohort.

Patients with higher IgM indices presented lower platelet counts compared to those with lower IgM levels, suggesting that a stronger IgM polyclonal‐mediated humoral immune response may be associated with more pronounced thrombocytopenia. Thrombocytopenia in dengue has been linked to platelet lysis and accelerated clearance, processes that immune mechanisms may mediate. IgM is a pentameric polyclonal immunoglobulin as opposed to high‐affinity DENV‐specific monoclonal IgG, with and neutralizing potential. Therefore, IgM could be associated to non or sub‐neutralizing anti‐DENV responses, which may ultimately contribute to disease severity. In particular, platelet‐associated IgM (PAIgM) has been reported to correlate with decreased platelet counts and disease severity, supporting the hypothesis that IgM may contribute directly to platelet destruction during DENV infection [31, 32, 33]. Additionally, the magnitude of IgM seroconversion observed at admission may serve as an early biomarker for aiding in the risk stratification of patients. However, in this cohort, no significant association was observed between IgM levels and final clinical classification or length of hospitalization, suggesting that while IgM may contribute to early hematological alterations, its role as a predictor of clinical outcomes remains uncertain. Further studies are warranted to clarify these potential interactions, including the precise mechanisms by which IgM may contribute to platelet lysis, the temporal relationship between humoral immune response and thrombocytopenia, and the potential impact on clinical outcomes.

In an integrated manner, the epidemiological, laboratory, and clinical findings of this study reflect the multifactorial complexity of the dengue epidemic in Minas Gerais in 2024. The predominance of DENV‐1, associated with a lower proportion of DENV‐2 and the reintroduction of DENV‐3, created a co‐circulation scenario that was reflected in the observed clinical manifestations. The high rate of IgG positivity indicates that a considerable portion of the patients likely had a secondary infection, which may have contributed to the more heterogeneous clinical profile. In this context, the laboratory parameters showed patterns consistent with the pathophysiology of the disease: thrombocytopenia predominated in most patients and was more pronounced in cases positive for DENV‐2, while the hematocrit remained relatively stable, possibly due to early clinical management. The association between higher IgM reactivity and lower platelet count suggests that the initial humoral response may be related to the observed hematological alterations, although without a direct impact on the final clinical classification. Taken together, these findings indicate that, although each isolated parameter contributes to the characterization of the disease, no single marker proved sufficient to independently predict severity. Thus, the data reinforce the importance of an integrated assessment involving serology, viral detection, and hematological monitoring to understand the clinical evolution of patients during large epidemics.

This study has limitations that should be considered when interpreting the results. Patient selection was influenced by the healthcare system strain during the outbreak, as hospitalization was based on clinical criteria rather than laboratory confirmation, potentially introducing selection bias. Moreover, clinical and laboratory data collection was not always standardized, leading to unequal numbers of observations across patients and varying follow‐up periods. Another limitation was the predominance of DENV‐1 cases over DENV‐2, which may have reduced the statistical power to detect differences between serotypes.

Despite these limitations, our findings emphasize the importance of continuous clinical and laboratory monitoring during dengue outbreaks, with particular attention to platelet counts as a key parameter for risk stratification. The observed correlation between serological and molecular results further highlights the need for integrated diagnostic strategies that account for the disease phase to improve accuracy. Notably, the differences associated with DENV‐2 underscore that epidemiological surveillance should consider not only viral circulation but also the potential impact of each serotype on clinical outcomes. These insights are particularly relevant in the context of the recent reintroduction of DENV‐3 in Brazil, which may influence future outbreak dynamics, clinical presentations, and healthcare preparedness strategies.

## 5 Conclusion

This study provides a comprehensive clinical, laboratory, and epidemiological characterization of the 2024 dengue outbreak in Minas Gerais, Brazil. While DENV‐1 predominated, DENV‐2 was associated with more pronounced thrombocytopenia, and the recent reemergence of DENV‐3 underscores the ongoing complexity of serotype co‐circulation. Despite differences in specific laboratory parameters, overall clinical progression was favorable under proper monitoring and supportive care. These findings highlight the importance of integrated diagnostic strategies, continuous clinical and laboratory surveillance, and targeted epidemiological monitoring to guide patient management and public health responses during dengue outbreaks.

## Author Contributions

Conceptualization: S.H.P., A.P.M.F.L., P.A.A. Methodology: S.H.P., A.P.M.F.L., C.T.N., G.O.V.C., P.A.A. Validation: S.H.P., A.P.M.F.L., A.M.C.N., V.A.A., S.H.C. Formal analysis: S.H.P., A.P.M.F.L., K.C.L.T., J.G.A.C.R., P.A.A. Investigation: S.H.P., A.P.M.F.L., A.M.C.N., V.A.A., S.H.C., K.C.L.T., J.G.A.C.R. Resources: J.G.A.C.R., A.T.C., F.G.F., P.A.A. Data Curation: S.H.P., G.O.V.C., A.M.C.N., V.A.A., S.H.C. Writing – Original Draft: S.H.P., P.A.A. Writing – Review and Editing: S.H.P., A.P.M.F.L., V.P.‐M., O.A.M.F., J.G.A.C.R., A.T.C., F.G.F., P.A.A. Funding acquisition: J.G.A.C.R., V.P.‐M., O.A.M.F., A.T.C., F.G.F., P.A.A.

## Ethics Statement

The investigation was approved by the *Ethics and Research Committee of René Rachou Research Center – Fiocruz Minas* (CAAE: 79060024.8.0000.5091).

## Conflicts of Interest

The authors declare no conflicts of interest.

## Supporting information


**Figure S1:** Annual confirmed dengue cases in Belo Horizonte city, Minas Gerais, Brazil, from 2014 to 2024. **Figure S2:** Annual distribution of dengue virus serotypes in Belo Horizonte city, Minas Gerais, Brazil, from 2014 to 2024.

## Data Availability

The data that support the findings of this study are openly available in SamilleHenriques/Paper‐DENV2024_BeloHorizonte_EMH at https://github.com/SamilleHenriques/Paper-DENV2024_BeloHorizonte_EMH.git.
