# Supplementary material for: Clinical, Serological, and Molecular Profile of Dengue Patients With Warning Signs During the 2024 Outbreak in Belo Horizonte, Brazil
Source: J Med Virol. 2026 Jan 15;98(1):e70805. doi: 10.1002/jmv.70805 (PMC12805315; doi:10.1002/jmv.70805)
Supplement: Supplementary file 1 — Figure S1: Annual confirmed dengue cases in Belo Horizonte city, Minas Gerais, Brazil, from 2014 to 2024. Figure S2: Annual distribution of dengue virus serotypes in Belo Horizonte city, Minas Gerais, Brazil, from 2014 to 2024. [file JMV-98-e70805-s001.docx]

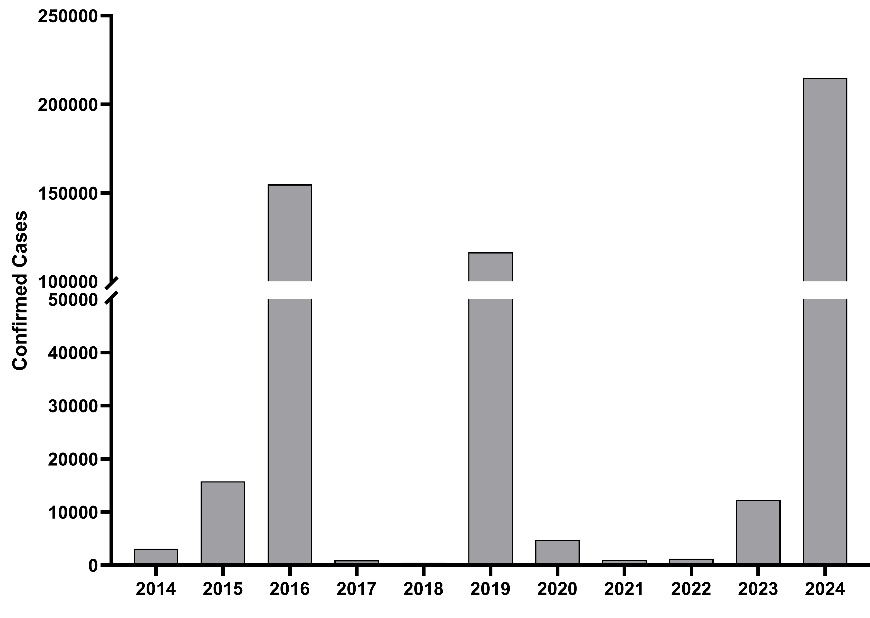


**Figure S1.** Annual confirmed dengue cases in Belo Horizonte city, Minas Gerais, Brazil, from 2014 to 2024. Bar chart depicting the number of laboratory-confirmed dengue cases reported annually in Belo Horizonte city over 11 years. The figure highlights the major epidemic years of 2016, 2019, and 2024, each marked by substantial surges in case notifications compared with inter-epidemic years, which show markedly lower incidence. A break in the y-axis is used to accommodate the large variability in case numbers, emphasizing the magnitude of epidemic peaks relative to baseline years. This temporal pattern reflects the cyclical nature of dengue transmission in the region and the occurrence of explosive outbreaks driven by serotype shifts and changes in population immunity. Data provided by the Municipal Health Secretariat of the Belo Horizonte City Government. Available at: <<https://drive.google.com/drive/folders/1THxa-PSoWf-Ca-defnpPp8tcY46-qn4c>>. Accessed on Nov 25, 2025.


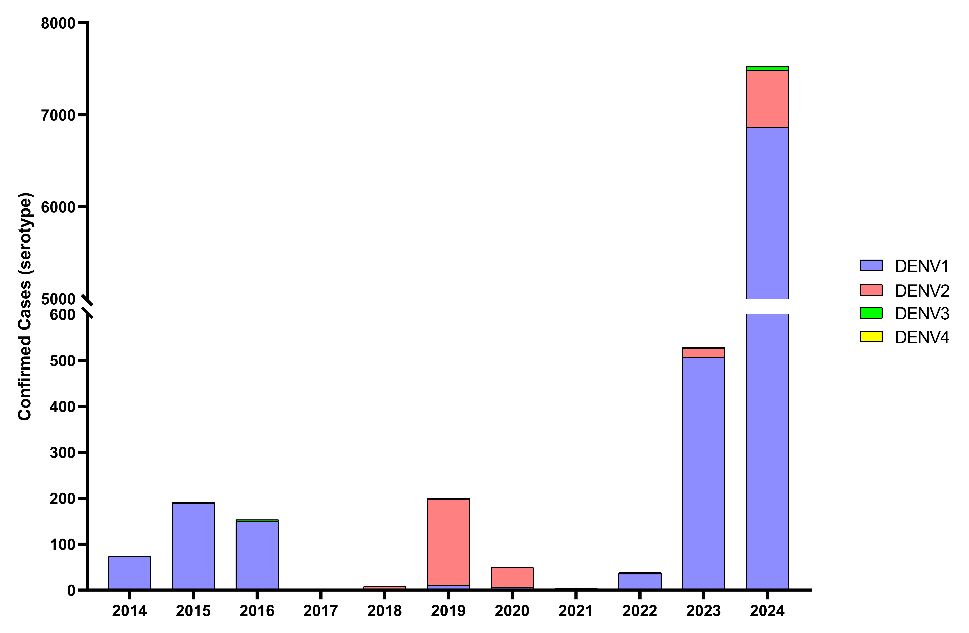


**Figure S2.** Annual distribution of dengue virus serotypes in Belo Horizonte city, Minas Gerais, Brazil, from 2014 to 2024. Stacked bar chart showing the yearly number of confirmed dengue cases stratified by DENV serotype (DENV-1, DENV-2, DENV-3, and DENV-4). The data highlight pronounced fluctuations in serotype circulation over the 11-year period. DENV-1 predominated throughout most years, including the major epidemic periods of 2016, 2023, and 2024, with markedly higher case counts in 2024. DENV-2 showed intermittent but notable peaks, particularly in 2019 and 2024, whereas DENV-3 appeared only at very low levels in 2024. No DENV-4 cases were reported during the entire period. A break in the y-axis is used to accommodate the large increase in serotype-specific case numbers during epidemic years. This temporal pattern illustrates shifts in serotype dominance that likely contributed to the intensity of recent outbreaks. Data provided by Brazilian Ministry of Health. DATASUS: TABNET – Epidemiological data. Available from: <<https://datasus.saude.gov.br/>>. Accessed on Nov 25, 2025.
